# Improvement of the reduction in catastrophic health expenditure in China’s public health insurance

**DOI:** 10.1371/journal.pone.0194915

**Published:** 2018-04-10

**Authors:** Dengfeng Wu, Fang Yu, Wei Nie

**Affiliations:** 1 Economics and Management School, Jiujiang University, Jiujiang City, China; 2 Jiujiang University Hospital, Jiujiang City, China; TNO, NETHERLANDS

## Abstract

This study aimed to locate the contributing factors of Catastrophic Health Expenditure (CHE), evaluate their impacts, and try to propose strategies for reducing the possibilities of CHE in the context of China’s current public health insurance system. The financial data of all hospitalization cases from a sample hospital in 2013 were gathered and used to determine the pattern of household medical costs. A simulation model was constructed based on China’s current public health insurance system to evaluate the financial burden for medical service on Chinese patients, as well as to calculate the possibilities of CHE. Then, by adjusting several parameters, suggestions were made for China’s health insurance system in order to reduce CHE. It’s found with China’s current public health insurance system, the financial aid that a patient may receive depends on whether he is from an urban or rural area and whether he is employed. Due to the different insurance policies and the wide income gap between urban and rural areas, rural residents are much more financially vulnerable during health crisis. The possibility of CHE can be more than 50% for low-income rural families. The CHE ratio can be dramatically lowered by applying different policies for different household income groups. It’s concluded the financial burden for medical services of Chinese patients is quite large currently, especially for those from rural areas. By referencing different healthcare policies in the world, applying different health insurance policies for different income groups can dramatically reduce the possibility of CHE in China.

## Introduction

### Background

Basic health care and universal health coverage are the main goals in many countries. In the last two decades, Chinese Government has been struggling at all levels to achieve this goal, and a nationwide health insurance system has been established, effectively lowering medical expenses and improving the availability of medical services. After a series of reforms, a Basic Health Insurance (BHI) system has been established in China, mainly consisting of three components, namely, Basic Health Insurance for Urban Employees (BHIUE), Basic Health Insurance for Urban Residents (BHIUR), and New Rural Cooperative Medical System (NRCMS). (Shown in Table 1).

**Table 1:** BHI system components in China.

BHI coverage	Insurant	Funding Sources
BHIUE	Urban employees	Insurant; Employer;
BHIUR	Urban non-employee residents	Insurant; Government
NRCMS	Rural residents	Insurant; Government

Besides BHI, a Critical Illness Insurance (CII) system, which is mainly funded by social pooling and the government, has also been launched to provide additional financial aid to patients suffering from extreme much hospitalization expense.

However, due to the huge income gap in China, a considerable part of families, especially those in the rural areas, still cannot afford necessary medical services. Families being dragged into poverty because of excessive medical costs have become serious social phenomena.

Catastrophic health expenditure (CHE) means that excessive proportion of a family’s income being used for necessary healthcare services which may infect other compulsory expenditure or even cause poverty. Currently, a threshold proportion of 40% has been widely used, especially by World Health Organization (WHO), which means that necessary healthcare expenditure of a family accounts for at least 40% of the household income after necessary food expenses.

According to a study from the Lancet published in 2012 [1], 12.9% of the families in China suffer from CHE. According to the data in the first half of 2015 from a hospital in Jiujiang City, the CHE ratio is 14.2% (CHE cases details shown in Table 2). The abovementioned statistics indicate that the current health insurance system has not fundamentally solved the problem of excessive medical expenses. Current health insurance policies should be further improved.

**Table 2:** Survey data of CHE in a metropolis sample hospital in 2015.

	Number of Cases	Yearly Household Income per capita(RMB)	Medical expense/(Income-food expense)	Average Medical Expenses(RMB)	Average Reimbusement Amount by BHI(RMB)	Average Reimbusement Amount by CII(RMB)	Average out-of-pocket Amount(RMB)
**Urban Patients**	2	14504	0.6132	32208	9349	2340	20519
**Rural Patients**	28	4589	0.9049	16990	4842	2151	9997
**Total**	30	5020	0.9258	18004	5142	2164	10698

One case has no insurance in 2 urban cases; One case has no insurance in 28 Rural cases

### Development of the health insurance system in China and literature review

The development of China’s health insurance system has undergone several stages. Before the health insurance reform in 1994, with limited coverage and national funding, the Chinese health insurance system only provide free healthcare service for employees in governmental and institutional organizations, as well as state-owned enterprises. Since 1994, BHIUE has been implemented. In July 2007, China State Council launched BHIUR to expand insurance coverage to include students, children, senior citizens, and other non-employees in urban areas. Simultaneously, NRCMS was implemented to provide medical insurance coverage for residents in rural areas. A nationwide framework of the BHI system was then established. Thereafter, the CII system was introduced in one region after another. In general, the implementation of BHI and CII has effectively reduced the financial burden on patients and played an important role in improving the availability of medical services in China.

Chinese scholars have launched a series of studies about the health insurance system and poverty. According to the analysis of Lu Hui (2012) [2] on the current health insurance policy in China’s rural areas, CHE has dropped by 11.67% and 19.05% in poor and non-poor groups respectively since the implementation of BHI, which indicated that the current health insurance system was in favor of the non-poor groups and showed certain tendency for reverse compensation. Lu Hui also pointed out that individual medical assistance and its coordination with NRCMS failed to lead a positive effect on the financial risk reduction for the rural poor groups. From the viewpoint of Zhang Yuanjie (2015) [3], although NRCMS aimed to improve the life quality and medical services in rural areas, especially to eliminate the possibility of poverty caused by serious diseases, significant part of rural families were still unable to gain effective financial support. According to the analysis of Hu Hongwei et al. (2012) [4], which was based on the data of nine cities, health insurance could raise the absolute amount of household medical spending and its relative ratio, whereas household medical spending would be significantly inhibited by poverty.

Internationally, scholars have provided valuable ideas on the elimination of CHE. Guy Carrin (2002) [5] introduced the practice of the Korean Government. In Korea, personal income ranking is the decisive factor of out-of-pocket costs for healthcare services, and the excess will be reimbursed by public medical institutions. For the top 20%, 20%-50% and the last 50% insured patients in the personal income ranking, the highest out-of-pocket amount will be 3,375 USD,2,531 USD and 1,688 USD, respectively. According to Guessous’ study of Switzerland (2012) [6], the cap amount of out-of-pocket costs is 726 USD, and the excess will be undertaken by the government.

The abovementioned studies revealed existing problems in China’s current health insurance policy and provided some useful theoretical references for future improvements. Based on the abovementioned conclusions, this study aims to achieve the following objectives:

1. To estimate the distribution of CHE cases (urban or rural, high income or low income) in China to find key problems and propose policy improvements;
2. To evaluate the effect on CHE by adjusting deductibles of current health insurance policies for different income groups;
3. To calculate the fiscal input needed by the government to avoid CHE.

Traditionally, CHE appraisal is based on sampling survey from inpatients in hospitals located in cities or towns, collecting data on their hospitalization expenses and family income. However, this method ignores the fact that some of the poorest people in China cannot afford to go to hospital even when they are heavily sick or seriously injured. They ask for informal treatment only for its low cost, such as from crude private clinics in rural areas. Therefore, accurate data on the traditional method can be difficult to obtain. A new method should be applied to the simulation for improved accuracy.

## Method

System Dynamics is suitable for solving complex social issues. Compared with other modeling methods, System Dynamics has the following characteristics: (1) suitable for solving long-term and periodical problems, (2) suitable for research with insufficient data, (3) suitable for handling complex social economic issues with moderate precision, and (4) conditional forecast [7–8].

In this study, based on the pattern of household income and hospitalization expense data in Jiangxi Province, System Dynamics and statistical theory will be used to establish a mathematical simulation model to assess the “pro-poor” effect of the current health insurance system. After patient payment is balanced with government financial capability, some proposals for current health insurance system will be made aiming to achieve maximum reduction of CHE.

The model will establish functions to simulate household income, medical expenses, insurance reimbursement, and patient out-of-pocket amount to assess pro-poor effect of current BHI, especially for low-income groups, and the reasonability of the system and possibility for future improvement.

According to the survey, the average number of new hospitalized cases in a typical metropolis sample hospital per day is 78.

Initial Time = 0, Final Time = 61 (61x128 = 7808 <8000), Time Step is set to 0.0078125, Time Unit = day, which means two months;

The modeling consists of three parts. The first part is the modeling of hospitalization expenses, mainly for simulating medical costs. The second part is the calculation of the out-of-pocket costs of patients based on current BHI and CII policies. The third part is the simulation of the household income of patients and to assess the frequency and ratio of CHE in the entire research period.

Modeling is based on the following assumptions:

1. Just chronic non-communicable diseases involved.
2. In each family, there is only one hospitalized patient each year.
3. Each patient has only one hospitalization record each year.
4. Outpatient costs are not considered in the calculation of CHE.
5. Social status, occupation, and background of the patients are not considered.
6. The effect of household income on medical expenses is not considered.

**Fig 1:**
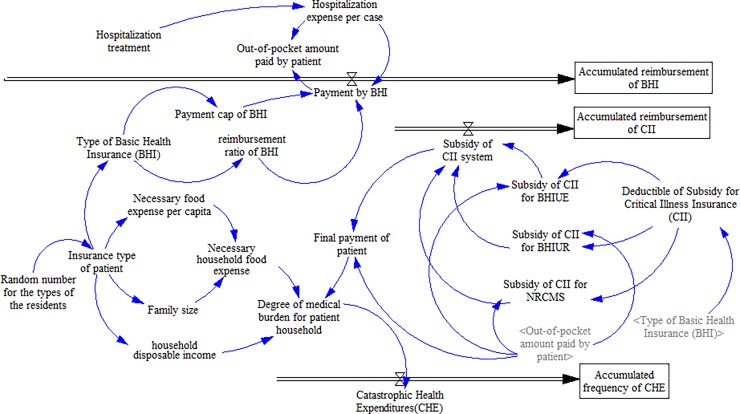
Flowchart for effect assessment for the public insurance system.

On the basis of the current health insurance system, a flowchart is established and shown in Fig 1.

### Simulation of hospitalization expenses

As an average of 78 daily hospitalization cases occurred in the sample hospital in 2013,

Hospitalizationtreatment=IFTHENELSE(RANDOMUNIFORM(1,128,1)<=78,1,0) (1)

The variables produced will have a value of 1 or 0 to represent “hospitalization” or “non-hospitalization” respectively. Uniform Random Distribution Function will adjust the number of daily hospitalization cases to 78.

The simulation of hospitalization expenses is one of the key points of modeling. According to the review of relevant studies, hospitalization expenses generally conform to lognormal distribution. By studying the subsidiary plan of NRCMS, Wang Ke (2000) [9] mentioned that hospitalization expenses can be fitted by lognormal distribution. Based on healthcare data of 37,054 residents in 11,577 households in Shanghai City, Jiangsu Province, and Shandong Province, Yanbing Zeng (2011) [7] attempted to fit hospitalization expenses into several distributions and found that lognormal distribution was the best in comparison with the others.

**Fig 2:**
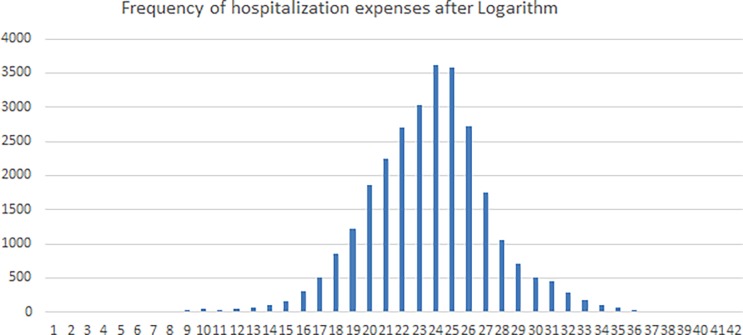
Frequency of hospitalization expenses after logarithm in the sample hospital in 2013.

In the survey, the frequency of the data-oriented hospitalization expenses in 2013 after logarithm with an interval of 0.25 is shown in Fig 2. The K-S test shows that it obeys normal distribution. Therefore, the model uses lognormal distribution to simulate hospitalization expenses.

Through the data collection of 28,411 hospitalization cases in the sample hospital in 2013, hospitalization expenses by department are shown in Table 3. The minimum, maximum, mean, and standard deviation were 79.84, 486686.45, 8002.13, and 12209.65 RMB, respectively; after their logarithms are taken, they will be 4.38, 13.0954, 8.5158, and 0.9632 RMB, respectively. They will then be used into the function as parameters.

**Table 3:** Hospitalization expenses by department in the sample hospital in 2013.

	Number of Cases	Minimum Hospitalization Expense(RMB)	Maximum Hospitalization Expense(RMB)	Average Hospitalization Expense(RMB)	Standard Deviation of Average Hospitalization Expense(RMB)
ICU	248	186.64	486686.45	28750.95	51787.2
Traditional Chinese Medicine	32	1130.01	15795.77	4996.28	2884.72
Breast Surgery	5	565.32	15262.6	6949.13	4976.98
Paediatrics	79	333.38	5822.1	2344.09	1335.67
Endocrinology	1233	127.9	60900.76	7894.3	5153.31
Stomatology	330	199.91	97752.5	7157.89	7533.8
Respiratory Medicine	1960	173.31	101698.48	7565.47	7501.4
Gynaecology and Obstetrics	1886	220.14	34160.71	4687.19	3911.52
Rehabilitation Medicine	146	131.9	177151.84	14122.71	24157.87
Thoracic Surgery	621	134.9	137684.6	10730.15	15271.7
Cardiovascular Medicine	2740	225	271608.33	8513.02	12459.64
Emergency	706	150.17	43871.84	3214.82	2615.05
Infectious Disease	1	1428.75	1428.75	1428.75	0
General Surgery	1772	115.5	131670.92	9601.15	11970.26
Urinary Surgery	960	231.9	48343.5	6928.39	6946.18
Gastroenterology	1986	138.72	71933.85	6338.98	5053.48
Pain management	433	375.41	51518.69	5821.77	4428.74
Dermatology	1734	117.9	63942.78	3043.67	2938.95
Ophthalmology	1538	122.5	37490.42	5220.38	3990.14
Neurology	1886	136.4	343587.3	9137.82	14792.86
Otolaryngological	1318	124.9	48310.65	5112.73	3356.82
Anorectal	191	107.5	14567.98	3695	1597.91
Hepatobiliary Surgery	1311	130.35	82826.34	10649.46	11465.52
Nephrology	1197	322.5	157114.2	7184.78	7110.93
Aural Surgery	432	237.18	280548.06	19881.56	32877.56
Hematology & Oncology	1227	675.11	106990.84	11651.7	12232.82
Rheumatism	270	127.74	59083.49	4760.05	5825.42
Orthopedics Ⅰ	624	230.9	140484.04	12108.38	13903.75
Orthopedics Ⅲ	383	94.2	70954.44	10429.84	11757.06
Orthopedics Ⅱ	741	140	281438.71	13717.25	21921.84
Bone Surgery	421	125.12	152150.07	10797.83	15146.8
Sum	28411	79.84	486686.45	8002.13	12209.65

$$\begin{array}{l} hospitalization\;expense\;per\;case\;\\ =INTEGER\left(EXP\left(RANDOM\;NORMAL\left(4.38,13.0954,8.5158,0.9632,2\right)\right)\;*\;Hospitalization\;treatment+0.5\right) \end{array}$$ (2)

As “*Hospitalization treatment*” has a value of 0 or 1, “hospitalization expense per case” is zero or non-zero. Only non-zero cases will be regarded as hospitalization that actually happens and included in the following simulation.

### Modeling for health insurance system

This model will be established based on current health insurance policy of Jiujiang City, Jiangxi Province (shown in Table 4).

**Table 4:** Reimbursement policy of health insurance in Jiujiang, Jiangxi Province.

	Yearly Reimburement Amount Maximum by BHI(RMB)	Nominal Reimbursement Ratio	CII Deductible(RMB)	CII Policy	Payment cap by CII (RMB)
BHIUE	60000	85%-90%	8000	***Step Subsidy Ratio Policy*:** 8000–10000, 30%; 10001–20000 40%; 20001 or above 50%;	190000
BHIUR	50000	50%-75%	5000	***Step Subsidy Ratio Policy*:** 5000–8000, 20%; 8000–20000 30%; 20001 or above 50%;	70000
NRCMS	80000	50%-75%	9000	***Step Subsidy Ratio Policy*:** 9001–59000, 50%; 59001–109000, 60%; 109001 or above 70%;	250000

BHI includes BHIUE, BHIUR, and NRCMS, which are represented by 1, 2, and 3, respectively. Numbers 4 and 5 are assigned to urban residents and rural residents without insurance, respectively.

$$\begin{array}{l} Type\;of\;Basic\;Health\;Insurance=IF\;THEN\;ELSE\\ \left(Patient\;status=1,1,IF\;THEN\;ELSE\left(Patient\;status=2,2,IF\;THEN\;ELSE\left(Patient\;status=3,3,IF\;THEN\;ELSE\left(Patient\;status=4,4,5\right)\right)\right)\right) \end{array}$$ (3)

The actual reimbursement ratio of three insurance coverages are different and lower than their respective nominal reimbursement ratio. Data collection in the sample hospital shows that they are about 70%, 50%, and 39%. For those without insurance, the reimbursement ratio is 0%.

$$\begin{array}{l} Payment\;percentage\;by\;BHI=IF\;THEN\;ELSE\\ \left(Type\;of\;Basic\;Health\;Insurance=1,0.7,IF\;THEN\;ELSE\left(Type\;of\;Basic\;Health\;Insurance=2,0.5,IF\;THEN\;ELSE\left(Type\;of\;Basic\;Health\;Insurance=3,0.39,0\right)\right)\right) \end{array}$$ (4)

PaymentofBHI=Hospitalizationexpensepercase*PaymentpercentagebyBHI (5)

$$\begin{array}{l} Out–of–pocket\;amount\;paid\;by\;patient\;\\ =\;INTEGER\left(\left(Hospitalization\;expense\;per\;case–Payment\;of\;BHI\right)*100\right)/100 \end{array}$$ (6)

The variable “Accumulated reimbursement of BHI” is a LEVEL variable in Vensim, and its initial value is 0.

In Table 4, CII deductibles are 8,000, 5,000, and 9,000 RMB for BHIUE, BHIUR, and NRCMS cases, respectively. In the model, the deductible for cases without insurance coverage is set to be 1×10^8^, which means no subsidy from health insurance.

DeductibleofCII=([(0,0)−(10,1e+008)],(1,8000),(2,5000),(3,9000),(4,1e+008),(5,1e+008)) (7)

Because of different health insurance coverages have different deductibles, the types of CII can be decided only by their respective deductibles.

$$\begin{array}{l} Subsidy\;of\;CII\;for\;BHIUE\;=\;INTEGER(MIN(IF\;THEN\;ELSE\\ ("Deductible\;of\;Subsidy\;for\;Critical\;Il\ln ess\;Insurance\;(CII)"=8000,IF\;THEN\;ELSE("Out‑of‑pocket\;amount\;paid\;by\;patient">=8000\\ :AND:"Out‑of‑pocket\;amount\;paid\;by\;patient"<10000,("Out‑of‑pocket\;amount\;paid\;by\;patient"‑8000)*0.3,\;\\ IF\;THEN\;ELSE("Out‑of‑pocket\;amount\;paid\;by\;patient">=10000\\ :AND:"Out‑of‑pocket\;amount\;paid\;by\;patient"<20000,600+("Out‑of‑pocket\;amount\;paid\;by\;patient"‑10000)*0.4,\\ IF\;THEN\;ELSE("Out‑of‑pocket\;amount\;paid\;by\;patient">=20000,4600+\\ \left("Out‑of‑pocket\;amount\;paid\;by\;patient"‑20000)*0.5,0))),0),190000)+0.5\right) \end{array}$$ (8)

$$\begin{array}{l} Subsidy\;of\;CII\;for\;BHIUR\;=\;INTEGER(MIN(IF\;THEN\;ELSE\\ ("Deductible\;of\;Subsidy\;for\;Critical\;Il\ln ess\;Insurance\;(CII)"=5000,IF\;THEN\;ELSE\\ ("Out‑of‑pocket\;amount\;paid\;by\;patient">=5000:AND:"Out‑of‑pocket\;amount\;paid\;by\;patient"<8000,\\ ("Out‑of‑pocket\;amount\;paid\;by\;patient"‑5000)*0.2,IF\;THEN\;ELSE("Out‑of‑pocket\;amount\;paid\;by\;patient">=8000\\ :AND:"Out‑of‑pocket\;amount\;paid\;by\;patient"<10000,600+("Out‑of‑pocket\;amount\;paid\;by\;patient"‑8000)*0.3,\\ IF\;THEN\;ELSE("Out‑of‑pocket\;amount\;paid\;by\;patient">=10000:AND:"Out‑of‑pocket\;amount\;paid\;by\;patient"<20000,\\ 1200+("Out‑of‑pocket\;amount\;paid\;by\;patient"‑10000)*0.4,IF\;THEN\;ELSE("Out‑of‑pocket\;amount\;paid\;by\;patient">=20000,\\ 5200+("Out‑of‑pocket\;amount\;paid\;by\;patient"‑20000)*0.5,0)))),0),70000)+0.5) \end{array}$$ (9)

$$\begin{array}{l} Subsidy\;of\;CII\;for\;NRCMS\;=\;INTEGER(MIN(IF\;THEN\;ELSE\\ ("Deductible\;of\;Subsidy\;for\;Critical\;Il\ln ess\;Insurance\;(CII)"=9000,IF\;THEN\;ELSE("Out‑of‑pocket\;amount\;paid\;by\;patient">=9000\\ :AND:"Out‑of‑pocket\;amount\;paid\;by\;patient"<59000,("Out‑of‑pocket\;amount\;paid\;by\;patient"‑9000)*0.5,\\ IF\;THEN\;ELSE("Out‑of‑pocket\;amount\;paid\;by\;patient">=59000:AND:"Out‑of‑pocket\;amount\;paid\;by\;patient"<109000,\\ 25000+("Out‑of‑pocket\;amount\;paid\;by\;patient"‑59000)*0.6,IF\;THEN\;ELSE("Out‑of‑pocket\;amount\;paid\;by\;patient">=109000,\\ 55000+("Out‑of‑pocket\;amount\;paid\;by\;patient"‑109000)*0.7,0))),0),250000)+0.5) \end{array}$$ (10)

In the aforementioned functions, medical subsidy can be calculated according to its CII type or considered 0 for non-insurance cases. Each case can only be categorized into one CII type at the most.

SubsidyofCIIsystem=SubsidyofCIIforBHIUE+SubsidyofCIIforBHIUR+SubsidyofCIIforNRCMS (11)

Out–of–pocketamountpaidbypatient=INTEGER((Hospitalizationexpensepercase–PaymentbyBHI)*100)/100 (12)

### Simulation of the household financial capacity for medical services

For the pattern of resident disposable income data, most scholars believed that income obeyed a skewed distribution and was influenced by regional economic development, economic traits, and fairness degree of income distribution. Statistically fitting income data properly has been challenging in academic research. Relative scholars proposed different fitting solutions, typically including Lognormal, Beta, Pareto, and Gamma and Weibull distribution.

Majumder and Chakravaty (1990) [10] found that the goodness-of-fit of Beta distribution was next only to a comprehensive fitting method devised by them for the data of personal income in the USA in 1960, 1969, and 1980. McDonald and Xu (1995) [11] pointed out that the degree of fitting of GB2 distribution came first among 11 fitting methods used to fit household income data in the USA. After studying developing countries, including India, Kenya, and Iraq, Z.W. Kmictowicz (1975) et al. and J.Rajaraman (1975) [12–13], found that the household income and household income per capita data exhibit characteristics of lognormal distribution. Ding Hua (1993) [14] also thought that lognormal distribution can fit the 1983–1988 rural income data in JiangSu Province very well.

In this research, by referencing family samples in 2013 in JiangXi Province, attempts were made to use Beta and lognormal distributions to fit urban and rural household income data of patients, and the results indicated that the goodness-of-fit of lognormal distribution was better than that of Beta. Thus, lognormal distribution was selected to fit urban and rural household income data. Simulated effects are compared with official data and shown in Table 5.

**Table 5:** Fitting household income by lognormal distribution.

	Urban					Rural				
	Low Income	Low-and-Medium Income	Medium Income	Medium-and-High Income	High Income	Low Income	Low-and-Medium Income	Medium Income	Medium-and-High Income	High Income
Average Household Income from Government Report (RMB)	41005	57803	63734	77951	103264	15717	25775	33476	44762	63815
Simulated Average Household Income (RMB)	41409	54769	66006	79065	107309	16089	24743	32869	43520	70070
Deviation	0.99%	-5.25%	3.56%	1.43%	3.92%	2.37%	-4.00%	-1.81%	-2.77%	9.80%

Data source: 2014 Jiangxi Statistical Yearbook

Relative official data on household income and expenses in 2013 are shown in Table 6. After the logarithm of the data is taken and the mean and standard deviation are calculated, the function is built and shown as follows:

**Table 6:** Data about family in 2013 in Jiangxi Province.

	Urban					Rural				
	Low Income	Low-and-Medium Income	Medium Income	Medium-and-High Income	High Income	Low Income	Low-and-Medium Income	Medium Income	Medium-and-High Income	High Income
Share of the Total (%)	20	20	20	20	20	20	20	20	20	20
Average Number of Family Members	3.67	3.32	2.9	2.83	2.48	5.13	4.52	4.11	3.97	3.36
Average Yearly Disposable Income per capita (RMB)	11172.97	17410.43	21977.32	27544.46	41638.73	3063.82	5702.36	8145.04	11275.06	18992.58
Average Household Income (RMB)	41004.80	57802.63	63734.23	77950.82	103264.05	15717.40	25774.67	33476.11	44761.99	63815.07
Average Food Expensed per capita (RMB)	3564.06	4564.48	5741.8	6569.16	7450.41	1639.23	2156.96	2394.92	2750.82	3360.75

Data source: 2014 Jiangxi Statistical Yearbook

According to the survey of Jiujiang City, more than 95% of the residents have been covered by BHI (BHIUE, 12.5%; BHIUR, 16.6%, NRCMS, 65.9%). The percentages of uninsured residents in urban and rural areas are 3.9% and 1.1%, respectively.

Randomnumberforthetypesoftheresidents=INTEGER(RANDOMUNIFORM(1,1000,1))

As mentioned in 3.2, five natural numbers, namely, 1, 2, 3, 4, and 5, are assigned to represent different health insurance statuses. In the function below, the patient numbers of different insurance statuses will be generated according to a certain ratio:

$$\begin{array}{l} Insurance\;type\;of\;patient\;\\ =\;\left(\left[\left(0,0\right)-\left(1000,10\right)\right],\;\left(1,1\right),\;\left(125,1\right),\;\left(126,2\right),\;\left(291,2\right),\left(292,3\right),\left(950,3\right),\left(951,4\right),\left(989,4\right),\left(990,5\right),\left(1000,5\right)\;\right) \end{array}$$

In addition, two types of necessary food expenses exist, namely, urban and rural, and their values are decided by the lowest food expenses of the urban and rural family group (The lowest food expenses required per capita are 3,564 RMB (about 600 USD) and 1,639 (about 280 USD) RMB per year for urban and rural residents, respectively, which are the official data from the local government in 2013). The modeling of family size (Average family sizes in urban and rural areas are 3.67 and 5.13 persons, respectively, according to the data from the province where the sample hospital is located.) and necessary food expenses per capita are shown as follows:

Necessaryhouseholdfoodexpense=Necessaryfoodexpensepercapita*Familysize

Familysize=([(0,0)−(10,10)],(1,3.67),(2,3.67),(3,5.13),(4,3.67),(5,5.13))

Necessaryfoodexpensepercapita=([(0,0)−(10,4000)],(1,3564),(2,3564),(3,1639.23),(4,3564),(5,1639))

According to the definition of CHE, a family’s compulsory healthcare spending accounts for more than 40% of the total household income besides necessary food expenses. In addition, a maximum value is used to avoid negative numbers.

$$\begin{array}{l} Ratio\;of\;the\;patients’\;medical\;burden\;\\ =\;actual\;payable\;amount\;of\;the\;patient\;/\;MAX\left(1,\left(household\;disposable\;income\;–\;household\;necessary\;food\;expenses\right)\right) \end{array}$$

CatastrophicHealthExpenditures(CHE)=IFTHENELSE(Ratioofthepatients’medicalburden>=0.4,1,0)

Accumulative CHE is a LEVEL variable, which counts the total CHE with time, and its initial value is 0.

## Analysis of the simulation outcomes

**Fig 3:**
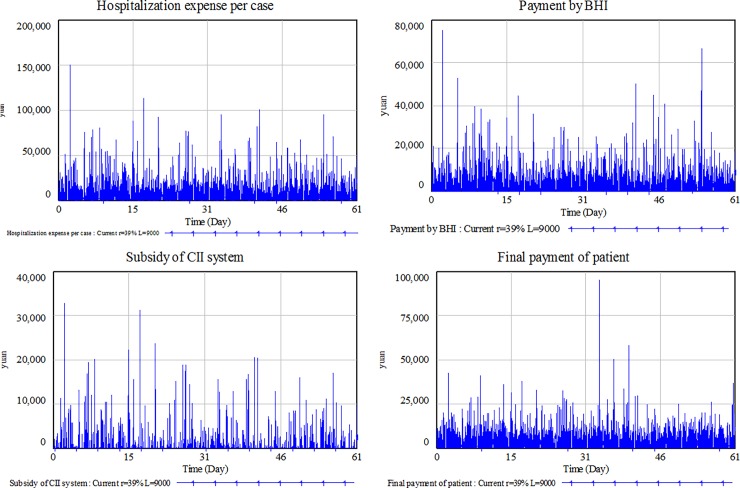
Simulation results of the model.

After running the Vensim simulation model, the reimbursement amount of BHI and CII, as well as the out-of-pocket costs of patients are demonstrated in Fig 3 and Table 7. In general, the medical insurance system has shown a positive effect on relieving patients’ financial burden for healthcare services, although a significant part of the total medical costs (about 53.16%) is still undertaken by the patients themselves.

**Table 7:** Share of patient out-of-pocket amount in hospitalization expense.

(Unit: RMB)	Hospitalization Expense	Reimbursement Amount by BHI	Reimbursement Amount by CII	out-of-pocket Amount
Average	7807	3282	375	4150
Total	37170161	15627682	1783645	19758834
Share of the Total	100%	42.04%	4.80%	53.16%

### Distribution of the CHE families

**Fig 4:**
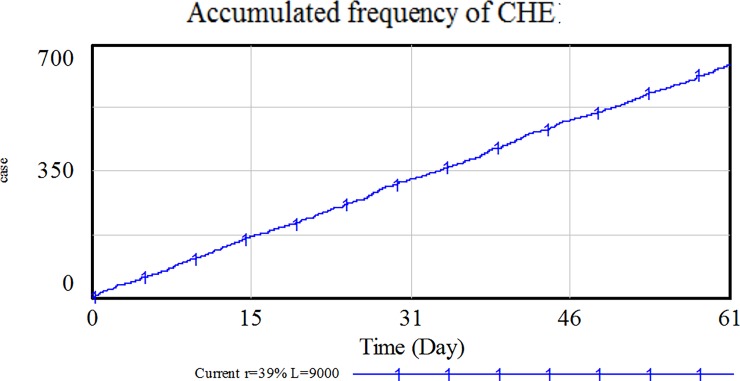
Number of CHEs in the simulation.

According to the simulation results, there are 643 CHE cases, as shown in Fig 4, accounting for 13.5% of the all 4,761 hospitalization cases, which is almost to 12.9% published by Lancet in 2011.

Most CHE cases are from rural families, 95% of which belong to NRCMS and non-insurance groups (shown in Table 8). The detailed data of CHE cases in each household income groups is listed in Table 9. In the 584 CHE cases with NRCMS coverage, 60% are from low-income group, and 85% are from low-income and low-and-medium-income groups. For urban families, no CHE cases were found in the high-income and medium-and-high-income groups.

**Table 8:** Distribution of CHE in different types of insurance.

	Number of CHE Families	Share of the Total
BHIUE	3	0.47%
BHIUR	9	1.40%
NRCMS	584	90.82%
Urban Non-insurance	25	3.89%
Rural Non-insurance	22	3.42%
SUM	643	100.00%

**Table 9:** Distribution of CHE in the insured group.

	Urban					Rural				
	Low Income	Low-and-Medium Income	Medium Income	Medium-and-High Income	High Income	Low Income	Low-and-Medium Income	Medium Income	Medium-and-High Income	High Income
Share of the Total	20%	20%	20%	20%	20%	20%	20%	20%	20%	20%
Average Household Income(RMB)	41444	54716	65344	77851	105314	15738	24307	32488	43320	69973
Average Reimburement Amount by Health Insurance(RMB)	3084	3545	3585	3379	3127	3417	3535	3409	3624	3272
Number of CHE Cases	8	3	1	0	0	352	151	60	17	4
CHE Ratio (%)	1.3%	0.5%	0.2%	0.0%	0.0%	59.1%	25.3%	10.1%	2.9%	0.7%

In summary, because of the significant income gap between rural and urban areas, current health insurance system shows very limited pro-poor tendency. The investment on NRCMS and efforts on relative policy-making must be increased.

### Simulation analysis of improvement effect of adjusting reimbursement ratio and deductible

As the majority of the CHE cases are NRCMS insurant, NRCMS should be the priority for adjustment.

**Fig 5:**
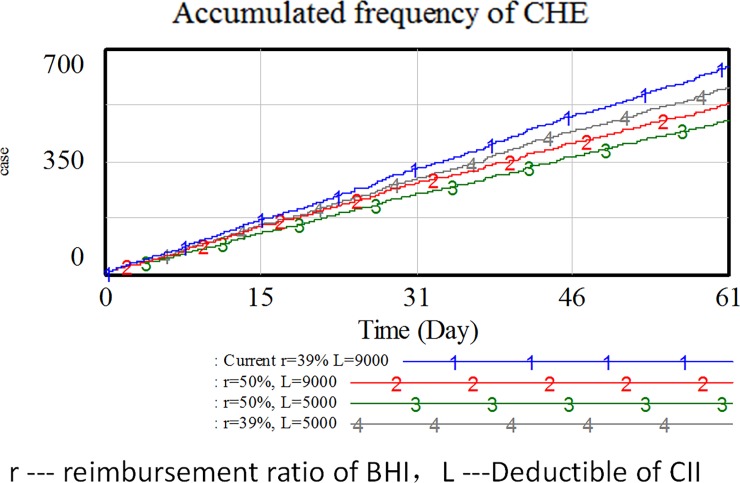
CHE cases in different reimbursement policies.

In the simulation model, NRCMS deductible and reimbursement ratio are adjusted separately and simultaneously. From results shown in Fig 5 and Table 10, when the combination of NRCMS deductible and subsidiary ratio is adjusted, the proportion of CHE can hardly be changed, whereas insurance costs significantly increase. Although the adjustment can reduce the number of CHE cases to a certain degree, no fundamental change is achieved.

**Table 10:** CHE change corresponding to insurance policy adjustment.

Insurance Policy	Current r = 39% L = 9000	r = 39%, L = 5000	r = 50%, L = 9000	r = 50%, L = 5000
Reimbursement Amount by BHI (RMB)	15627682	15627682	18344722	18344722
Reimbursement Amount by CII (RMB)	1783645	2997668	1262932	2148854
Total Reimbusement (RMB)	17411327	18625350	19607654	20493576
Reimbusement Increase %	-	6.97%	12.61%	17.70%
Number of CHE Cases	643	580	529	477
Percentage of CHE	13.51%	12.18%	11.11%	10.02%
Percentage Vary of CHE	-	-1.32%	-2.39%	-3.49%

The main reason lies in the low level of household income of the medium and low-income groups in rural areas. The average income per year of the low-income and medium-and-low-income groups is 15,717 and 25,775 RMB, respectively; after the deduction of necessary food expenses, the remaining income is 7,308 and 16,026 RMB, respectively. According to the threshold of CHE, the endurable out-of-pocket costs are 2,932 and 6,410 RMB, much lower than the NRCMS deductible. Thus, these families can hardly avoid CHE even with the financial aid of NRCMS.

### Solution of setting a cap amount of medical expenses for different income groups in China

According to international practices, there are three strategies being implemented to avoid CHE.

Strategy A, which has been implemented in Thailand and India[15], refers to almost free medical services in public hospitals.

Strategy B, having been implemented in South Korea and Switzerland[5][6], refers to a cap amount policy depending on personal income ranking. Medical costs exceeding the cap amount will be undertaken by the government.

Strategy C, which is typically found in some states of the USA, refers to the shared responsibility of medical costs by the government and individuals, but a copayment will not exceed the financial capability of the patient.

Due to the large population, both strategy B and C are quotable experiences for China.

#### The design and simulation of strategy B for China: Five-level Cap Amount Policy

According to this policy, a five-level system will be established depending on the family income ranking, and different maximum out-of-pocket amount will be assigned to each income level. Although this policy is easy to understand and implement, it requires massive investment, and CHE will be unavoidable for the families of lowest income in each group. By simulation in the model, the cap amounts for each level are shown in Table 11.

**Table 11:** Calculation of out-of-pocket cap based on five-level family income.

	Urban					Rural				
	Low Income	Low-and-Medium Income	Medium Income	Medium-and-High Income	High Income	Low Income	Low-and-Medium Income	Medium Income	Medium-and-High Income	High Income
1. Share of the Total(%)	20	20	20	20	20	20	20	20	20	20
2. Average Number of Family Members	3.67	3.32	2.9	2.83	2.48	5.13	4.52	4.11	3.97	3.36
3. Household Income per capita(RMB per Year)	11172.97	17410.4	21977.3	27544.46	41638.73	3063.82	5702.36	8145.04	11275.06	18992.58
4. Necessary Food Expenses per capita(RMB per Year)	3564.06	3564.06	3564.06	3564.06	3564.06	1639.32	1639.32	1639.32	1639.32	1639.32
5. Household Incom—Necessary Food Expenses(RMB per Year)	27925	45970	53398	67865	94425	7308	18365	26739	38254	58307
6. Affordable Household Medical Expenses(RMB per Year)	11170	18388	21359	27146	37770	2923	7346	10696	15302	23323
7. Reimbursement Amount by BHI and CII(RMB per Year)	5121.5	5121.5	5121.5	5121.5	5121.5	2770.5	2770.5	2770.5	2770.5	2770.5
8.Item 6 + Item 7(RMB per Year)	16291.5	23509.5	26480.5	32267.5	42891.5	5693.5	10116.5	13466.5	18072.5	26093.5
9. Proposed Cap Amount for Medical Services	16500	23500	26500	32500	43000	5500	10000	13500	18000	26000

According to the simulated data, the total payable amounts of medical services in urban and rural areas are 6,364,126 and 11,060,597 RMB, respectively. (shown in Table 12).

**Table 12:** Extra investment needed for five-level cap amount policy.

`	Urban						Rural					
	Low Income	Low-and-Medium Income	Medium Income	Medium-and-High Income	High Income	Sum	Low Income	Low-and-Medium Income	Medium Income	Medium-and-High Income	High Income	Sum
Family number %	20%	20%	20%	20%	20%	100%	20%	20%	20%	20%	20%	100%
Increased Investment Needed for Health Insurance (RMB)	259100	325810	181540	94740	63340	924530	2460333	1501810	1031350	818300	453970	6265763
Increase Ratio	4.07%	5.12%	2.85%	1.49%	1.00%	14.53%	22.2%	13.6%	9.3%	7.4%	4.1%	56.6%

The total nationwide reimbursement amount paid by BHIUE and BHIUR was 680.1 billion RMB in 2013. To eliminate CHE in urban areas, an extra investment of 98.95 billion RMB will be needed.

Based on the governmental investment standard of NRCMS in 2013 (280 RMB per capita), another 158.5 RMB per capita (127.5 billion RMB, total) would be needed to eliminate CHE of rural residents in China.

#### Design and simulation of Strategy C for China: Cap amount policy of individual cases

According to this policy, the maximum out-of-pocket cost for healthcare service of a patient will not exceed 40% of his family income deduct basic living expenses. This policy may fully eliminate CHE because it is based on individual cases, but the administrative cost will be very high as family income will have to be verified depending on the case.

According to simulated data, the total payable amounts of medical services in urban and rural areas are 67,307 and 2,351,031 RMB, respectively. Increased percentages based on initial medical financial investment are 1.06% and 21.3% (shown in Table 13), which means an extra investment of 7.22 billion RMB will be needed for the 573.22 million urban insurants of BHIUE and BHIUR, and 65 billion RMB for the 805 million rural insurants of NRCMS in China.

**Table 13:** Extra investment needed for cap amount policy of individual cases.

	Urban						Rural					
	Low Income	Low-and-Medium Income	Medium Income	Medium-and-High Income	High Income	Sum	Low Income	Low-and-Medium Income	Medium Income	Medium-and-High Income	High Income	Sum
Family number %	20%	20%	20%	20%	20%	100%	20%	20%	20%	20%	20%	100%
Increased Investment Needed for Health Insurance (RMB)	29823	36572	912	0	0	67307	1380325	602029.6	248432.4	102426.8	17816.4	2351031
Increase Ratio	0.47%	0.57%	0.01%	0.00%	0.00%	1.06%	12.5%	5.4%	2.2%	0.9%	0.2%	21.3%

## Conclusion and discussion

The most recent global studies on assessing and reducing CHE involved hundreds of papers. Wagstaff (2018) [16] estimated impoverishment in 122 countries using 516 surveys between 1984 and 2015, considering Out-of-pocket spending on health could add to the poverty head count and the depth of poverty by diverting household spending from non-health budget items. Increasing the share of total health expenditure that was prepaid, especially through taxes and mandatory contributions, could help reduce CHE. Jan (2016) [17] believed CHE still incurred in Asia area even insurance offered some protection based on wide survey and logistics regression models. Xie(2018) [18], Si(2017) [19], Liu(2017) [20], Gu(2017) [21], Yang(2016) [22], Qin(2017) [23], Mao(2017) [24] demonstrated financing inequity on health expenditure for different group in China and concluded Chinese government needed to invest more fund to expand further Social Health Insurance Programs for low-income household especially in rural area to avoid CHE. The research of Sahu (2017) [25], Gwatidzo(2017) [26], Pandey(2017) [27], Pradhan(2017) [28], Nandi(2017) [29] showed Out-of-pocket expenditure (OOPE) was an obstacle in the path of getting universal health coverage in India and the proportion of households experiencing catastrophic health expenditure in India had increased in past years. Mohanty (2017) [30] suggested increasing government spending on health and increasing households' access to health insurance could reduce catastrophic health spending and multidimensional poverty based on 9247 households sample size in Myanmar, Nepal and India. Rahman(2018) [31] suggested to reform health financing systems of Bangladesh to avoid high dependency on out-of-pocket spending, otherwise the incidence of CHE would increase from 17% to 20% in 5 years by estimation of Bayesian regression model. Rad(2017) [32], Rezapour(2018)[33] considered there was a high degree of CHE in Iran especially caused by chronic non-communicable diseases or in retirees based on sample survey. Aji(2017) [34] affirmed positive effect of Indonesian Health Care Financing System on incidence of CHE. By panel dataset analysis, it’s found the relationship between improvements of health care financing performance and the existing health reform demonstrated a mutual reinforcement, which should be maintained to promote equity and fairness. Based on 5610 sample size and Panel dataset analysis, Lee(2016) [35] found households with a person with a disability faced higher catastrophic health expenditures. Exemption or reduction of out-of-pocket payments in the National Health Insurance and additional financial support were needed. Scott(2017) [36–37]argued over 7 of 10 uninsured patients would be at risk of CHE even in United States on the analysis of 579,683 and 189,059 sample size. Diaz-Gonzalez(2017) [38], Amaya-lara(2016)[39], Kockaya(2017) [40] researched samples in Mexico, Colombian, Costa Rican respectively. Their researches indicated health policy was critical for vulnerable groups against financial risk and reduce the incidence of catastrophic healthcare spending. In Africa, Anderson(2017) [41],Subramanian(2018) [42], Ngcamphalala(2018) [43], Mchenga(2017) [44], Aregbeshola(2017) [45], Akazili(2017) [46], Rickard(2017) [47], Edoka(2017) [48] confirmed incidence of CHE was higher than expectation. There was a need to address the burden created by direct out-of-pocket payments to move towards achieving universal health coverage.

The above studies show that CHE is a global problem not only in low- and middle-income countries, but also in high-income countries. The medical insurance policy, especially the proportion and amount of patient out-of-pocket expenditure is the most important factor in determining the incidence of CHE. This paper is devoted to the improvement of China's medical insurance policy. Compared with the above studies, this study mainly made the following expansion.

1. Most of the above studies data come from in-patient sample survey in hospital. But in this paper, data of Family income is from government published Authoritative data covering all classes of society in China which can better cover extreme poverty cases that the patients normally just seek treatment in clinic instead of hospital or refuse to see doctor even having serious illness. It improves accuracy of CHE assessment.
2. Typical research methods of above studies are regression analysis, statistical analysis or panel analysis. This paper demonstrates method to reduce CHE incidence through the simulation modeling of System Dynamics. Compared with other methods, simulation model can obtain a dataset of each household for further research. Especially it can assess the effect of new insurance policy quickly and conveniently when adjusting reimbursement ratio or deductible line.

The following conclusions were drawn from this study.

1. In the current health insurance system, subsidies for various income groups show minimal differences, and limited support is given for low-income families. Pro-poor Effect in the current system is obviously insufficient, especially for rural residents.
2. More than 90% of CHE cases came from NRCMS families, residents in rural area in China, indicating that NRCMS policies must be further improved.
3. The adjustment of subsidiary ratio and deductible level in small dimensions that the government is attempting to implement can only result in a limited positive effect on CHE.
4. Cap amount policy can eliminate CHE thoroughly, although it needs heavy investment by the government. For a five-level cap amount policy, a total investment of 127.5 billion RMB (20.24 billion US dollars) or 160 RMB per capita (about 25 US dollars per capita) is necessary. For the cap amount policy of individual cases, a total investment of 65 billion RMB or 82 RMB per capita is needed.

We do not think that the method applied in this study is perfect because it is based on a series of hypotheses and information symmetry[49]. However, it provides a special channel and solution to view CHE and current insurance policy in China. In future, research on this topic can be planned to focus on the following fields:

1) Whether a health insurance fund planned across the country instead of a province is effective;

We think it’s more effective, but we need more data from different provinces to support our opinion, especially based on mathematical calculation.

2) The effect of an individual’s action on CHE, such as the manner in which individuals borrow money from each other;

It’s interesting idea raised by peers, which will involve the process of Game Theory and psychological decision-making. This research will help solve CHE problem from both macro and micro aspects.

3) Whether the government can permit poor patients to pay their medical expenses on an installment basis and whether the improvement has a positive or negative effect on the government and patient.

In USA, paying medical expenses on installment is a common solution for poor people. However it’s based on a relatively sound social credit system. In China social credit system needs to be further improved. Thus the solution to paying on installment will involve cross-disciplinary knowledge rather than just mathematical model.

The above problems will be gradually solved in the future research. The reform of health care policy involves the happiness of China's huge population, so it deserves more research from related scholars all over the world.

## Supporting information

S1 File CHE case details by survey in sample hospital. (XLSX)

S2 File Original medical expense data collected from sample hospital. (XLSX)

S3 File Simulation data about che. (XLSX)

S4 File Simulation model program of System Dynamic. (MDL)

S5 File Text of program. (TXT)
