# Correction: Improvement of the reduction in catastrophic health expenditure in China's public health insurance

**DOI:** 10.1371/journal.pone.0213243

**Published:** 2019-02-27

**Authors:** Dengfeng Wu, Fang Yu, Wei Nie

There is an error in the Funding section. The complete, correct funding information is: This research is funded by the National Natural Foundation of China 71661018, 71663034, CMB16-261, Project of Science and Technology Department 20161BBA10031.
